# Implementing a robust adverse event of special interest surveillance for novel oral polio vaccine type 2 rollout, Nigeria, March-July 2021

**DOI:** 10.11604/pamj.supp.2023.45.2.40228

**Published:** 2023-07-14

**Authors:** Samuel Luka Abbott, Sume Gerald Etapelong, Saheed Gidado, Kabir Yusuf Mawashi, Aboyowa Arayuwa Edukugho, Abdullahi Walla Hamisu, Abba Shehu, Elizabeth Adedire, Isiaka Ayodeji Hassan, Ndadilnasiya Endie Waziri, Omotayo Bolu, Usman Saidu Adamu

**Affiliations:** 1 African Field Epidemiology Network, Abuja, Nigeria,; 2 World Health Organization, Abuja, Nigeria,; 3 National Primary Health Care Development Agency, Abuja, Nigeria,; 4 United States Centers for Disease Control and Prevention Nigeria Office, Abuja, Nigeria

**Keywords:** Novel oral polio vaccine type 2, adverse event, immunization, AESI surveillance, Nigeria

## Abstract

**Introduction:**

novel oral poliovirus vaccine type 2 (nOPV2), designed to be more genetically stable than Sabin-strain oral poliovirus vaccine type 2 (mOPV2), is a new and key component of the Global Polio Eradication Initiative’s strategy to combat outbreaks of circulating vaccine-derived poliovirus type 2 (cVDPV2). The World Health Organization´s (WHO´s) emergency use listing (EUL) requires extensive safety monitoring for Adverse Event of Special Interest (AESI) in its use. We implemented AESI active surveillance to monitor the safety of the nOPV2 in Nigeria

**Methods:**

a cross-sectional assessment was conducted in Nigeria during March-June 2021 in 117 local government areas (LGAs) across 6 states and the Federal Capital Area with confirmed cVDPV2 transmission. We conducted active searches for nOPV2 AESI in all health facilities. Suspected events were ascertained, and vaccination and clinical data abstracted. Events were classified using WHO causality assessment algorithm. Data were analyzed using Epi info7.

**Results:**

total of 234 adverse events were reported after 21,997,300 doses of nOPV2 were administered, giving a crude reported incidence of 1 in 94,000 doses of nOPV2. Altogether, 221 of the 234 (94%) adverse events were classified. For 166 AESI ascertained to occur following a dose of nOPV2, the corrected crude incidence rate was 1 in 133,000 doses; 4 of the adverse events, were classified as consistent with casual association with nOPV2 vaccination.

**Conclusion:**

we found that nOPV2 had a low incidence of AESI following nOPV2 campaigns and no new or unexpected adverse event was reported. Safety monitoring should be sustained for early detection of signals and uncommon adverse events.

## Introduction

Nigeria, along with all countries in the World Health Organization African Region (WHO AFR) achieved wild poliovirus (WPV)-free status from the African Regional Certification Commission (ARCC) on 25 August 2020 [1]. However, transmission of circulating vaccine-derived poliovirus type 2 (cVDPV2) has persisted due to low population immunity against type 2 poliovirus resulting from low uptake of the routine immunization [2-5] and suboptimal quality of polio supplementary immunization activities (SIAs). In 2020, the Global Polio Eradication Initiative (GPEI) launched a new strategy to combat outbreaks of cVDPV2 with the novel oral poliovirus vaccine type 2 (nOPV2) [6]. The nOPV2 is genetically modified to be more stable than Sabin-strain oral poliovirus vaccine type 2 (mOPV2) and therefore less likely to revert to neurovirulence [4]. Furthermore, nOPV2 provides similar intestinal immunity to the Sabin OPV2 [7]. Currently, nOPV2 has received World Health Organization (WHO) Emergency Use Listing (EUL) recommendation for use in outbreak response [6]. Use of nOPV2 under EUL requires meeting specific mandatory criteria. Key among these criteria is highly sensitive surveillance for poliovirus (i.e., acute flaccid paralysis (AFP) and environmental surveillance) and Adverse Event Following Immunization (AEFI) [8]. In clinical trials, no serious adverse events (AE) were reported to be causally related to vaccination with nOPV2 [7]. Regardless, enhanced nOPV2 safety monitoring is required to facilitate early detection, investigation, and analysis of AEFIs and Adverse Events of Special Interest (AESIs) to ensure an appropriate and rapid evaluation.

An AESI is a pre-specified, medically significant event that has the potential to be casually associated with a vaccine product and needs to be carefully monitored and confirmed by special studies [9]. Global Polio Eradication Initiative (GPEI) partners proposed anaphylaxis, aseptic meningitis, acute disseminated encephalomyelitis, Guillain Barré Syndrome, transverse myelitis and other causes of AFP (i.e. poliomyelitis and vaccine associated paralytic poliomyelitis (VAPP)), and unexplained death as conditions for nOPV2 AESI active surveillance [10]. In February 2021, Nigeria obtained approval from GPEI to use nOPV2 under EUL after meeting the 21 indicators targets in the readiness checklist [6]. In March 2021, nOPV2 was deployed in a mass campaign in response to an outbreak of cVDPV2 across seven states. With this deployment, Nigeria became the first country to use nOPV2 in cVDPV2 outbreak responses [11]. To monitor the safety of nOPV2 in a campaign setting, the Government of Nigeria, with support of GPEI and in-country PEI partners, instituted active AESI surveillance in the states implementing cVDPV2 outbreak responses. The AESI active surveillance is expected to complement passive AEFI surveillance system and overall poliovirus surveillance to facilitate early detection and response to vaccine safety signals and AESI within a reasonable risk period following immunization with nOPV2. This paper describes the roll-out and implementation of the nOPV2 AESI surveillance in Nigeria, highlights the rate of AE among children vaccinated and proportion of AE that were casually related to nOPV2.

## Methods

**Figure 1:**
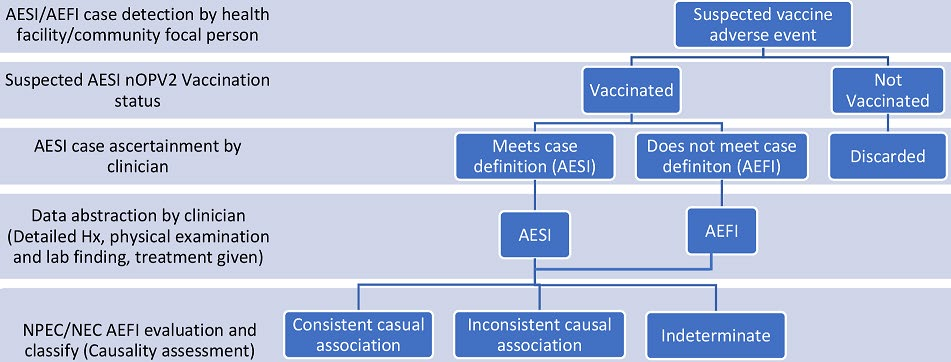
algorithm for adverse event identification and classification, nOPV2 AESI surveillance, Nigeria, March - July 2021

**Study participants and area:** this was a cross-sectional assessment conducted during March-July 2021 in 117 (15.1%) of the 774 Local Government Areas (LGAs) in Nigeria. We intensified surveillance for AESI and other AE among children 0-59 months after they received the nOPV2 during a 4-day, house-to-house vaccination campaign. During the 12 weeks period of surveillance intensification, trained senior health experts drawn from Governmental and GPEI partners visited health facilities across the 117 implementing LGAs to identify and collect data on suspected nOPV2 AE cases for review and classification Figure 1 shows the algorithm of the assessment.

**Development of guidelines and training modules:** the National Polio Emergency Operations Centre (EOC) National Surveillance Working Group (NSWG) developed nOPV2 AESI active surveillance guidelines and operational level-specific training modules. At the health facilities, the training modules focused on AESI detection and reporting using simplified AESI case definitions of signs and symptoms. An active AESI surveillance checklist was developed to be used by Disease Surveillance and Notification Officers (DSNOs) during visits to health facilities to systematically review health records for AESI that met the case definition. A supervisory checklist for state teams (government and partners) was also developed. The state level training modules covered the entire process of active nOPV2 AESI surveillance, including case detection, reporting, ascertainment, data abstraction, and investigation of AEFI and AESI. Global Polio Eradication Initiative adverse event of special interest (GPEI AESI) guidelines and training modules were adapted to suit local context [10].

**Training on nOPV2 AESI surveillance and AEFI causality assessment:** personnel were trained at different levels of the health system to ensure mastery of the concept of AESI surveillance.

**Training of National Expert Committee on AEFI:** members of the National Expert Committee on Adverse Event Following Immunization (NEC AEFI) were trained on nOPV2 AESI surveillance and causality assessment. The NEC AEFI consisted of pediatricians, pathologists, epidemiologists, public health physicians, laboratory scientists and immunization specialists. We used training materials adapted from WHO causality assessment methodology and GPEI nOPV2 AESI guidelines for the training.

**Training of state surveillance officers:** state level surveillance officers and GPEI partner staff from the 36 states and FCT were trained to build national capacity for nOPV2 AESI surveillance in preparedness for the use of nOPV2 vaccine beyond the six states with confirmed cVDVP2 outbreak. The participants were surveillance officers from state ministry of health and in-country PEI partners. Members of NSWG facilitated the training.

**Training of LGA surveillance officers:** cascaded training of LGA surveillance workforce from government and GPEI partners was conducted in all the implementing states. State surveillance officers supported by NSWG facilitated the training.

**Training of health workers at the facility:** health workers (surveillance focal person) in all health facility in the 117 LGAs were trained on nOPV2 AESI active surveillance. The training was facilitated by the LGA surveillance officers, supported by the state and NSWG officers.

**Training of clinicians:** the State and NSWG officers trained selected clinicians from implementing LGAs on nOPV2 AESI surveillance, case ascertainment and data abstraction for suspected AESI.

**Health facility-based active surveillance for nOPV2 AESI:** active surveillance for nOPV2 AESI commenced on the first day of nOPV2 use and implemented for 12 weeks after the last nOPV2 dose administered in the LGA. We used the Brighton´s Collaboration case definition to describe these nOPV2 AESI [12]. The health facility surveillance focal person used a simplified AESI case detection guide which has a combination of sign and symptoms of selected AESI (Table 1) during review of outpatient and inpatient registers in relevant units of the facility on a daily basis to facilitate detection and reporting of suspected nOPV2 AESI cases. The LGA surveillance officers conducted weekly surveillance visits to all designated high-priority surveillance focal sites and reviewed registers to identify suspected cases of AESI. All sites sent weekly reports of suspected cases or “zero report forms”. The state level officers conducted at least once monthly supportive supervision to the LGAs and health facilities to provide surveillance oversight and mentor surveillance officers for improved surveillance performance. The state officers supervised the LGA surveillance officers and at least two health facilities with the LGA surveillance officers.

**Table 1:** simplified WHO AFRO nOPV2 AESIΩ detection guide based on combination of signs and symptoms, Nigeria March 13 - July 7, 2021

Targeted AESI	X = Likelihood 1	Y = likelihood 2	Z = likelihood 3
**Group anaphylaxis**			
C1-anaphylactic reaction	X-suffocation/choking	E-difficulty in breathing	D1-generalized itching
	N-face-throat oedema	K-rash/urticaria	E1-generalized tingling sensation
	Y-generalized oedema	A1-hypotension	F1-red, itchy eyes
	Z-loss of consciousness/syncope	B1-tachycardia	G1-persistent dry cough
		C1-cold extremities	A-abdominal pain
			D-diarrhoea
			Q-vomiting
**Group meningo-encephalitis**			
c2-aseptic meningitis	B-coma	F-difficulty closing one eye	A-abdominal pain
c2-encephalitis	C-confusion	I-headaches	D-diarrhoea
c3-acute disseminated encephalomyelitis (ADEM)	H-neck stiff, neck/pain	J-lethargy	Q-vomiting
	L-convulsion/contracture	M-light sensitivity	G-fever
		H1-fontanelle bulging	
		I1-agitation	
		O-motor incoordination	
**Group paralysis**			
c4-Guillain Barre Syndrome	R-motor weakness or numbness	K1-decreased muscle tone	
c4- Fisher syndrome	J1-paralysis of one or two limbs	U-muscle atrophy	
c5-myelitis/transverse myelitis	O1- AFP	L1-muscle pain	
c7-vaccine-associated paralytic		N1-walking difficulty	
Poliomyelitis (VAPP) c8 - Vaccine Derived Polio Virus (VDPV)			
**Group death**			
c6-unexplained death	P-death		
Any child aged 0-59 months, presenting during the surveillance period with the following sign(s)/symptom(s) or in combinations: 1 sign/symptom in column X, or 2 signs/symptoms in column Y or 3 signs/symptoms in column Z or 1 sign/symptom in column Y + 2 signs/symptoms in column Z (1Y + 2Z)			
O1- AFP any child under 15 years of age with acute (sudden) onset of weakness or floppiness of one or more limbs or any person of any age with paralytic illness in whom a clinician suspects poliomyelitis (NB: for this AESI surveillance line list all AFP cases 0-59 months/)			
**WHO AFRO**: World Health Organisation Africa region, nOPV2: novel oral poliovirus vaccine type 2, ASESI: adverse event of special interest, AFP: acute flaccid paralysis			

**Data collection:** data were collected using six instruments: AESI reporting form, line listing form, ascertainment form, case-specific data abstraction form [10], AEFI investigation form [13] and AFP case investigation form [14]. We used the AESI reporting form for immediate reporting of suspected AESI regardless of their vaccination status regarding clinical presentation, socio-demographic information, vaccination history, investigation, diagnosis, and treatment of the suspected AESI. This information was summarized in the line-listing form for each case. The clinician used the ascertainment form to ascertain that the suspected AESI met the case definition of AESI condition. Detailed clinical and laboratory finding of ascertained cases were collected in the case specific abstraction form. The standard WHO AFR AFP case investigation form was used to collect AFP case-related data, and the AEFI investigation form to conduct a detailed investigation of every suspected AESI case vaccinated with nOPV2. All the data were collected in hard copies, maintained at the health facility, LGA and State levels. Scanned copies of completed data tools were emailed weekly to the national level.

**Data management:** the health facility shared completed AESI data forms with the LGA DSNO. The LGA DSNO compiled all data forms from all health facilities in the LGA and shared with the State surveillance officers. The state surveillance officers compiled all completed data forms from all the LGAs in the state and shared with the NSWG on Friday of every week. At the national level, designated surveillance/data officers reviewed the reports for completeness, collated reports from the states, and performed data analysis. Data were analyzed using EPI Info 7 [15] and results were presented as frequencies and proportions.

**Causality assessment: is the systematic review of data about an AEFI case with th**e aim to determine the likelihood of a causal association with the vaccine(s) received. The expert committee conducted causality assessment quarterly using the WHO causality assessment checklist and algorithm [16]. They ensured the AE satisfied the minimum criteria for causality assessment by establishing exposure to nOPV2 and a valid diagnosis for the adverse event. The checklist was used to systematically review the relevant and available information to gather evidence for possible causal association between the suspected AE and the nOPV2. An algorithm was used to determine the nature of relationship between the AE and nOPV2 using the evidence available. The association between the vaccine and AE was classified into one of three association groups, namely: consistent with casual association, inconsistent with causal association (coincidental) or indeterminate [16].

**Ethical considerations:** this assessment was an important activity of the National Polio EOC, which was implemented in line with the recommendations of the GPEI and the WHO for nOPV2 use. The protocol and tools were reviewed and approved by National Drug and Food Administration Agency of Nigeria, the regulatory body for medicinal and vaccine products in Nigeria.

## Results

**Training on nOPV2 AESI surveillance:** overall, 8,023 personnel were trained on AESI surveillance at the national level and in the cVDPV2 outbreak states. Altogether, 7,772 of 8023 (97%) were LGA and health facilities´ staff in the 6 cVDPV2 outbreak states and FCT while 251 (3%) where NEC AEFI members and State surveillance officers in all 36 states and FCT. Among 7,772 trained in the outbreak states, 515 (7.0%) were doctors, 313 (4.0%) were DSNOs, and 6,943 (89.0%) were community health extension workers (Table 2).

**Table 2:** health personnel trained for nOPV2 AESIΩ surveillance, March 13 - July 17, 2021, Nigeria

	No. (%) of health personnel				
State	+LGA	*DSNO/ADSNO	Medical doctors	Community health workers	Total trained
Bayelsa	8 (6.6%)	24(7.7%)	40(7.8%)	406(5.8%)	470(6.0%)
Delta	25(20.5%)	59(18.8%)	210(40.8%)	932(13.4%)	1201(15.6%)
FCT	6(4.9%)	22(7.0%)	43(8.3%)	566(8.2%)	631(8.1%)
Kebbi	21(17.2%)	60(19.2%)	71(13.8%)	1713(24.7%)	1844(23.7%)
Niger	25(20.5%)	54(17.3%)	40(7.8%)	1169(16.8%)	1263(16.3%)
Sokoto	23(18.9%)	38(12.1%)	61(11.8%)	1016(14.6&)	1115(14.3%)
Zamfara	14(11.5%)	56(17.9%)	50(9.7%)	1141(16.4%)	1248(16.1%)
Total	122	313	515	6943	7772

* Disease surveillance and notification officer/assistant disease surveillance and notification officer +local government area Ωnovel oral polio vaccine type 2 adverse event of special interest

**nOPV2 AESI surveillance visits:** local government areas DSNOs and their assistants conducted 12,977 (71%) of the expected 18,348 weekly active surveillance visits to the facilities. The missed visits were to low-priority sites, although weekly zero report forms were received from all sites including sites not visited during the week. Altogether, they visited 1,592 surveillance sites in the study area. The highest proportion (100%) of active surveillance visits was achieved in Zamfara State while the lowest (38%) was in the FCT area council.

**Suspected nOPV2 adverse events:** during the AESI active surveillance period, 234 suspected AE were reported for 21,997,300 doses of nOPV2 administered, giving a crude reported AESI incidence of 1.0 per 94,000 doses of nOPV2 (Table 3). Two hundred and twenty-nine (97.9%) of the AE were detected through the active AESI surveillance visits and the remaining 5 (2.1%) were reported to the facility by caregivers. Of the AE, 138 (58.7%) occurred in males; the median age was 25 months (range 2- 84 months). A total of 221 (94.4%) of the 234 AE were ascertained as suspected nOPV2 AESI by medical officers at subnational level. Of the 221 ascertained AESI, 219 (99.1%) had weakness of one or more limbs and investigated according to the GPEI guideline for investigating suspected AFP cases; adequate stool specimens were collected appropriately and tested in a WHO-accredited polio laboratory. Testing of the paired-stool specimens isolated 40 (18.2%) non-polio enteroviruses, 24 (11%) nOPV2, 1 (0.5%) Sabin 3, 1 (0.5%) cVDVP2 and 153 (69.9%) were had no isolate. We conducted 60-days follow-up examination to check for residual paralysis for 24 cases with nOPV2 stool-isolates. Notably, of the 234 AE reported as nOPV2-associated, the DSNO investigation determined that 220 (93.6%) occurred in children actually vaccinated with nOPV2 and of those, only for 166 (74.8%) did vaccination occur before symptoms onset.

**Table 3:** number of suspected nOPV2 AESIΩ reported by state, March 13 - July 7 2021, Nigeria

No (%) of AESI					
State	Number reported AESI	Number vaccinated with nOPV2	#Number ascertained as AESI	Number with symptom onset after vaccination	Number AESI abstracted
Bayelsa	34	30 (88.2%)	29 (85.3%)	23 (67.5%)	7 (20.6%)
Delta	15	11 (73.3%)	15 (100%)	10 (66.7%)	-
FCT	2	2 (100%)	2 (100%)	2 (100%)	-
Kebbi	128	127 (99.2%)	128 (100.0%)	114 (89.1%)	5 (3.9%)
Niger	18	15 (3.3%)	18 (100.0%)	11 (61.1%)	-
Sokoto	22	22 (100%)	22 (100%)	8 (36.4%)	-
Zamfara	15	15 (100%)	15 (100%)	11 (73.3%)	-
Total	234	223 (95.3%)	229 (97.9%)	179 (76.5%)	12 (5.1%)

#Number ascertained are total number of suspected cases presented to a medical doctor for review to ensure cases meet criteria for reporting as suspected AESI ΩNovel oral polio vaccine type 2 adverse event of special interest, FCT: federal capital territory

**Causality assessment:** altogether, 221 (94.0%) of the 234 suspected nOPV2 AE were presented to the NEC AEFI and the National Polio Expert Committee (NPEC) for review and classification. Thirteen were not presented due to insufficient information resulting from incomplete investigations occasioned by insecurity in settlements where these cases occurred. The expert committees reviewed 221 AE and assigned a likely diagnosis based on the clinical information available (Table 4). Of the 219 AE reported and investigated as AFP, 175 (79.2%) met the AFP case definition and 44 (20.1%) were not true AFP (non-AFP; not meeting the AFP case definition to exclude known conditions). The diagnosis of some of the non-AFP after review included severe malaria (n=9), cerebral palsy (3), Vaso-occlusive crisis in sickle cell patients (2), severe protein energy malnutrition (3) and acute diarrhoeal disease (4). The diagnosis for nOPV2 AESI with AFP included 48 transverse myelitis, 1 GBS, 3 VAPP, 1 cVDPV2 and 4 encephalitis (Table 4). The committees determined the eligibility of AE by establishing that vaccination with nOPV2 preceded AE for each case. A total of 54 (24.4%) of 221 were ineligible for classification because they either were not vaccinated or the AE occurred before their vaccination with nOPV2 based on the history taken during DSNO investigation; 34 (61.8%) of the ineligible AESI cases met the AFP case definition and 1 was indeterminate. In all, 166 (75.1%) of the 221 suspected AE reviewed were classification. The NEC AEFI determined that 4 of the 166 eligible AESI (2.4%) had consistent causal association with nOPV2 vaccination, 161 (97.0%) had inconsistent causal association to nOPV2 vaccination (coincidental due to underlying or emerging condition independent of the vaccination) and one (0.6%) lacked sufficient information for classification (Table 5). The AE with consistent causal association were 1 anaphylaxis and 3 VAPP cases; all four were reported from Kebbi State.

**Table 4:** NEC AEFI and NPEC diagnosis of suspected AESI, nOPV2 AESI surveillance, March 13 - July 7 2021, Nigeria

Targeted AESI	NEC AEFI/NPEC diagnosis of AE	N (%)
Group anaphylaxis	Anaphylaxis	1(0.5%)
Group meningo-encephalitis	Focal encephalitis	4 (1.8%)
Group paralysis	Myelitis/transverse myelitis	48 (17.6%)
	Guillain Barre syndrome	1 (0.9%)
	Circulating Vaccine-Derived Polio Virus (cVDVP2)	1(0.5%)
	Vaccine-Associated Paralytic Poliomyelitis	3 (0.5%)
	Traumatic/Injection neuritis	43 (24.3%)
	Monoplegia due to nonspecific viral infection	40 (20.7%)
	Hemiparesis/hemiplegia due to CNS infection	35 (14.4%)
	Electrolyte imbalance	5 (2.3%)
Others*	AEFI	40 (16.7%)
Total		221

* Cerebral palsy, malaria, acute diarrhoeal disease, measles, bronchopneumonia, seizure disorder, cellulitis, failure to thrive, protein-energy malnutrition, rickets, sepsis, vaso-occlusive crisis in sickle cell patient, hemorrhagic fever, lower limb trauma, muscular dystrophy, delayed developmental milestone, and spinal cord compression ΩNovel oral polio vaccine type 2 adverse event of special interest

**Table 5:** causality assessment summary of the suspected nOPV2 AESIΩ/AEFI¥ by State, March 13 - July 7, 2021, Nigeria

Classification	Bayelsa n (%)	Delta n (%)	FCT n (%)	Kebbi n (%)	Niger n (%)	Sokoto n (%)	Zamfara n (%)	National n
Ineligible	6 (9.4)	4 (7.6)	0	18 (34.0)	6 (11.3)	14 (26.4)	6 (11.3)	54
Consistent causal association to nOPV2 immunization	0	0	0	4 (100)	0	0	0	4
Inconsistent causal association to nOPV2 immunization	17 (11.3)	10 (6.3)	2 (1.3)	105 (63.8)	12 (7.5)	7 (4.4)	9 (5.6)	162
Indeterminate	0	0	0	0	0	1 (100)	0	1
Total	23 (10.7)	14 (6.7)	2 (0.9)	127 (56.9)	18 (8)	22 (10.2)	15 (6.7)	221

ΩNovel oral polio vaccine type 2 adverse event of special interest ¥Adverse event following immunization FCT: federal capital territory

Adverse event of special interest with consistent causal association with nOPV2 vaccine

**Case 1: anaphylaxis:** ZB, a 36-month-old female presented with rash, difficulty in breathing, abdominal pain, and diarrhea on 15/4/2021 about 30 minutes after receiving nOPV2 vaccine on 15/4/2021. No previous history of similar illness or significant medical history prior to the illness. The child was treated and made full recovery. The medical doctor ascertained the case as aphylactic reaction based on the clinical presentation and time interval between vaccine administration and symptoms onset. The NEC AEFI and NPEC made a diagnosis of anaphylaxis. With available evidence, the committees concluded that the classification is consistent with causal association to immunization based on time of onset after vaccination (A1 vaccine product related reaction as per published literature) [17,18].

**Case 2: vaccine associated paralytic poliomyelitis:** PA, a 24-month-old female, presented on May 20, 2021, with fever, weakness of the right leg which began on 11/5/2021. There was no trauma, fall or injection to the affected limb prior to onset of weakness. The child is fully vaccinated and received SIA nOPV2 on 16/4/2021. The child was treated with analgesics and multivitamins. Stool specimens testing isolated nOPV2 and the child had residual paralysis at 60-day follow-up examination. The NEC AEFI and NPEC made a diagnosis of vaccine-associated paralytic poliomyelitis. With the available evidence, the committee concluded that the classification was consistent with causal association with immunization (Vaccine product related reaction) The case meets the minimum criteria for a recipient VAPP as the child presented with clinically compatible poliomyelitis with onset 25 days after receiving nOPV2, residual paralysis 60 days after paralysis onset and stool specimens testing in WHO-accredited lab isolated nOPV2 but negative for WPV/VDPV.

**Case 3: vaccine associated paralytic poliomyelitis:** US, a 48-month-old female, presented on May 23, 2021, with history of fever, and weakness of the right lower limb which started on 12/5/2021. There was no trauma or injection prior to the onset of the limb weakness. The child had received 7 doses of OPV from polio campaigns; she was vaccinated with 2 doses of nOPV2 on 15/4/2021 and 22/5/2021. No previous history of hospitalization, allergy to previous vaccination, food or drug and no similar event in the past. The vaccine was maintained in good condition and there was no error in dosage or its administration. The child was ascertained as acute flaccid paralysis. Stool specimens testing isolated nOPV2. The child had residual paralysis at 60-day follow-up examination. The NEC on AEFI and NPEC made a diagnosis of vaccine-associated paralytic poliomyelitis. With the available evidence, the committee concluded that the classification was consistent with causal association with immunization (Vaccine product-related reaction). The case met the minimum criteria for a recipient VAPP as the child presented with clinically compatible poliomyelitis with onset 25 days after receiving nOPV2, residual paralysis 60 days after paralysis onset and stool specimens testing in WHO-accredited lab isolated nOPV2 but negative for WPV/VDPV.

**Case 4: vaccine associated paralytic poliomyelitis:** PS a 23-month-old female, presented with fever and an inability to walk due to weakness of right lower limb which started on 11/5/2021. There was no trauma or injection prior to the onset of the limb weakness. The child had received 9 doses of OPV (5 from polio SIA, 4 from routine immunization) and 1 dose of IPV. The last dose of OPV received was nOPV2, administered on 15/4/2021. No previous history of hospitalization, allergy to previous vaccination, food or drug and no similar event in the past. Vaccine was maintained in good condition and there was no error in dosage or its administration. A musculoskeletal system examination showed reduced tone, power and reflex in the affected limb. All other limbs were normal. The case was ascertained as acute flaccid paralysis. Stool specimen testing isolated nOPV2 and the child had residual paralysis Sixty-day after paralysis onset. The NEC on AEFI and NPEC made a diagnosis of vaccine-associated paralytic poliomyelitis. With the available evidence, the committee concluded that the classification was consistent with causal association with immunization (vaccine product related reaction). The case met the minimum criteria for a recipient VAPP as the child presented with clinically compatible poliomyelitis with onset 25 days after receiving nOPV2, residual paralysis 60 days after paralysis onset, and stool specimens testing in WHO-accredited lab isolated nOPV2 but negative for WPV/VDPV.

**Adverse event of special interest not consistent with causal association with nOPV2 vaccine:** cVDPV2: GS, a 38-month-old male child, presented with fever and weakness of the right leg on 3/5/2021. The child was vaccinated with a dose of nOPV2 on 13/4/2021 and developed fever on 22/April/2021. He received intramuscular injections in both buttocks on account of fever prior to onset of his symptoms on 26/4/2021. The child had no hospitalization, allergy to previous vaccination, food, or drug. Examination of the right leg revealed, reduced power (1/5), muscle tone and deep tendon reflex but intact sensation. All other limbs were normal. Stool specimen testing isolated cVDVP2 of the ZAS-1 emergence group, one of the four circulating strains being responded to with nOPV2. The physician made a provisional diagnosis of right-sided monoplegia probably due to paralytic polio. The NEC AEFI and NPEC made a diagnosis of Poliomyelitis due to cVDVP2. With available evidence, the committees concluded that the classification was inconsistent with causal association to immunization and was coincidental. The limb paralysis was due to cVDVP2 infection and not nOPV2 vaccination.

## Discussion

In this paper, we present the findings of an active surveillance activity to monitor the safety of nOPV2 and provide safety data to enable the relaxation of mandatory criteria for use of nOPV2 under EUL. We found that nOPV2 vaccine had a very low incidence of AE in children aged 0-59 months during mass vaccination campaigns. Of the 234 AE reported from 21,997,300 doses of nOPV2 administered in the 117 LGAs, 166 were determined to occur after administration of nOPV2. A low overall adjusted crude incidence of serious AE of one to 133,000 doses indicates the safety of the vaccines outside controlled clinical settings and is similar to what was reported among children aged 1-4 years and infants aged 18-22 months who received nOPV2 in clinical trials [7]. In the clinical trials, no causally-associated serious event or important medical event was documented as AE following immunization were majorly mild or moderate. As the country with first initial use globally, this assessment has generated safety data outside clinical trial settings, and among infants below 18 months old. Forty-five (23.1%) of the 234 suspected AE occurred in children below 18 months of age, and none of these AE was ultimately classified as causally related to the use of nOPV2.

National Expert Committee on Adverse Event Following Immunization and NPEC reviewed 221 ascertained AE of 234 reported AE and assessed potential causality. The majority (81.4%) of the 221 ascertained AE were acute flaccid paralysis. Of the 221 AE reported, 166 (75.1%) nOPV2 vaccination preceded onset of AE. Among the 166 AE detected that followed nOPV2 administration, 4 had consistent causal association with immunization with nOPV2 (vaccine product related reaction) after detailed review of available information and classification by the expert committee: a case of anaphylaxis and three vaccine-associated paralytic poliomyelitis cases [17,18]. This is an observed rate of one of these outcomes out of 10 million doses. OPV vaccination in a very rare instance may cause paralysis in recipients of vaccines or their contacts, a condition known as VAPP [19,20]. The finding of probable causal association between nOPV2 with both VAPP and anaphylaxis is not unexpected; VAPP is a documented risk of Sabin-strain OPV and anaphylaxis has been reported after OPV combined with other vaccines. Furthermore, the VAPP incidence from our study is what can be expected [20]. In Kebbi State, where the 3 cases of VAPP were reported from approximately 3.5 million doses of nOPV2 that were administered, the incidence of VAPP in the state was similar to 1 case in 2.7 million doses documented with the use of previous Sabin-strain OPV [21]. No other VAPP was found following 18.5 million doses administered in the other states and FCT area council. The association of AESI with nOPV2 found in our study may not have been expected with a vaccine genetically more stable than Sabin OPV. It remains judicious to continue to monitor the safety of the nOPV2 through active AESI surveillance and routine AEFI surveillance as the use of nOPV2 transitions to wider use in more countries with cVDPV2 outbreak under less stringent EUL requirement than in the initial use phase [22].

The majority of the nOPV2 AESI reported were AFP cases and very few of the other nOPV2 AESI like aseptic meningitis, acute disseminated encephalomyelitis, anaphylaxis, and encephalitis were documented. The high number of reported AFP cases without laboratory evidence of poliovirus infection and without residual paralysis is likely due to a high background incidence of transient non-polio enterovirus infections, injection neuropathy and neurologic illnesses like GBS that are detected by a robust and functional AFP surveillance system as in Nigeria. AFP is a clinical syndrome with obvious weakness of the one or more limbs that is detected and reported both through the hospital and community-based AFP surveillance system. As reported, approximately 20% of reported AFP were not true AFP and represented other diagnosable neurologic complications. Having national level training in which 237 state surveillance officers from 36 states and FCT were trained regardless of cVDPV2 outbreak status was in anticipation of the spread of the virus over time. As such, whenever a new state was infected, we proceeded directly with state and LGA level training. This approach ensured that additional nOPV2 AESI surveillance was promptly implemented during outbreak response SIAs in newly infected areas.

We recognized some limitations in this intervention. First, our study was conducted in a setting with minimal or no access to laboratory and radiological facility for confirmation of diagnosis, except for AFP. Laboratory workup for non-AFP AE might have been suboptimal, as such the diagnostic certainty largely depends on clinical judgement of clinicians. Secondly, there may have been underreporting of the non-AFP AESI because of the suboptimal laboratory capacity and lack of funding to conduct confirmatory investigation of the non-AFP AESI. Collection of cerebrospinal fluid by spinal tap is uncommonly conducted in Nigeria, and so aseptic meningitis cases could have been substantially undetected. Thirdly, insecurity in some settlements in some LGAs in 4 of the 7 states where the nOPV2 AESI surveillance was implemented affected free movement of people and resulted in limited access to and investigation of suspected AE in these insecure settlements.

## Conclusion

We found that nOPV2 vaccine has a reassuring safety profile and no new or unexpected safety concern was detected in field setting. Three VAPP among 21 million doses administered is within expected occurrence rates for oral polio vaccines. We recommend continuous safety monitoring through active surveillance as nOPV2 becomes more widely available for SIA response to cVDPV2 outbreaks.

**Disclaimer:** the findings and conclusions in this report are those of the authors and do not necessarily represent the official position of the U.S. Centers for Disease Control and Prevention.

### What is known about this topic

- Supportive supervision is a valuable programmatic strategy used to impart knowledge and help improve the skills of health workers in implementing their tasks and advance health outcomes in African countries;
- Open Data Kit (ODK) is an open-source mobile technology application that has gained popularity in recent years and is becoming widely used to capture and manage data in various public health programs or projects, especially in low-resource settings or low-to-middle-income countries.

### What this study adds

- Detailed comparative advantages of using ODK technology over paper forms to report supportive supervision conducted during polio Supplementary Immunization Activities (SIA) in Nigeria;
- The impact of adopting ODK to strengthen supervision reporting, coverage, and consequentially quality of supervision to guide vaccination teams in the service-delivery of oral poliovirus vaccine during polio SIAs in Nigeria.
